# Supplementary material for: Effect of Shading on Physiological Attributes and Proteomic Analysis of Tea during Low Temperatures
Source: Plants (Basel). 2023 Dec 24;13(1):63. doi: 10.3390/plants13010063 (PMC10780538; doi:10.3390/plants13010063)
Supplement: Supplementary file 1 [file plants-13-00063-s001.zip › Figure S1.pdf]

Figure S1: Primers used in tis study.

Gene sequence number	Primer name	Sequence (5'to3')	TM
Internal reference	18S-F	CAACCATAAACGATGCCGA	60
	18S-R	AGCCTTGCGACCATACTCC	
1	TEA_026230-F	GCTCATATTGGAGAGGCTAT	60
	TEA_026230-R	CAACAGGAAACGCAATGAA	
2	TEA_020688-F	CGGTCTTGAATGTGGTCAT	60
	TEA_020688-R	GAAAGGCGACTGGTTGTA	
3	TEA_023893-F	ATCAGGCAGTTGAACCAGA	60
	TEA_023893-R	TGAGGATGAGTGTCTTCTTGT	
4	NW_021026906-F	AGTGTGGAAGATGCCAAGTA	60
	NW_021026906-R	TTGTCTCACCAGCACCAT	
5	NW_021026072-F	AGTGTGGAGTCTCGTTCAG	60
	NW_021026072-R	GCAACCAAACCCAGCATAG	
6	NW_021024901-F	GTGAAACCTTTGGCTTTGTAGA	60
	NW_021024901-R	GGTGAGGAAGAGTCTTGGAA	
