# Supplementary figures and images for: Effect of Shading on Physiological Attributes and Proteomic Analysis of Tea during Low Temperatures
Source: Plants (Basel). 2023 Dec 24;13(1):63. doi: 10.3390/plants13010063 (PMC10780538; doi:10.3390/plants13010063)

Figure S2

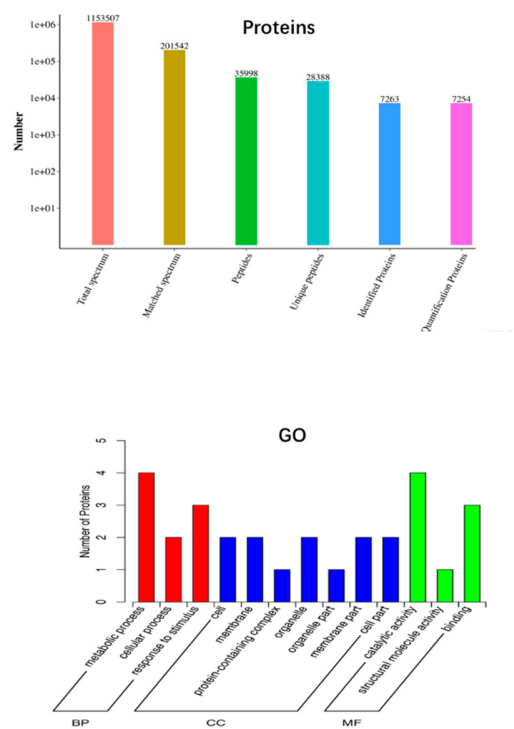

Supplement: Supplementary file 1 [file plants-13-00063-s001.zip › Figure S2.pdf]

Figure S3

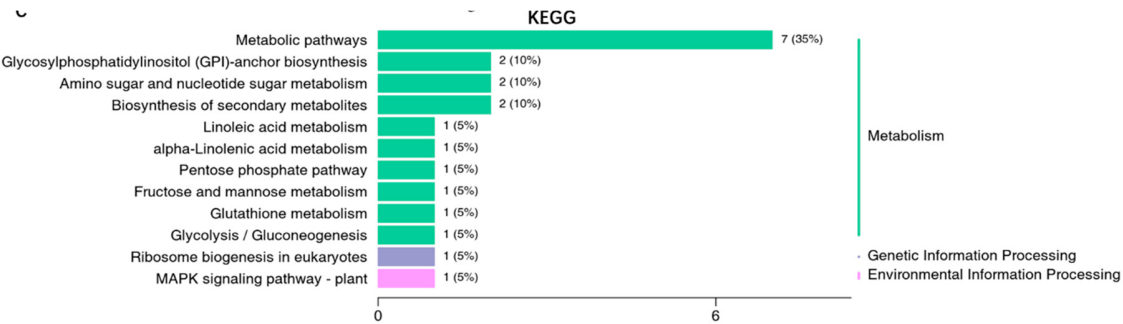

Supplement: Supplementary file 1 [file plants-13-00063-s001.zip › Figure S3.pdf]

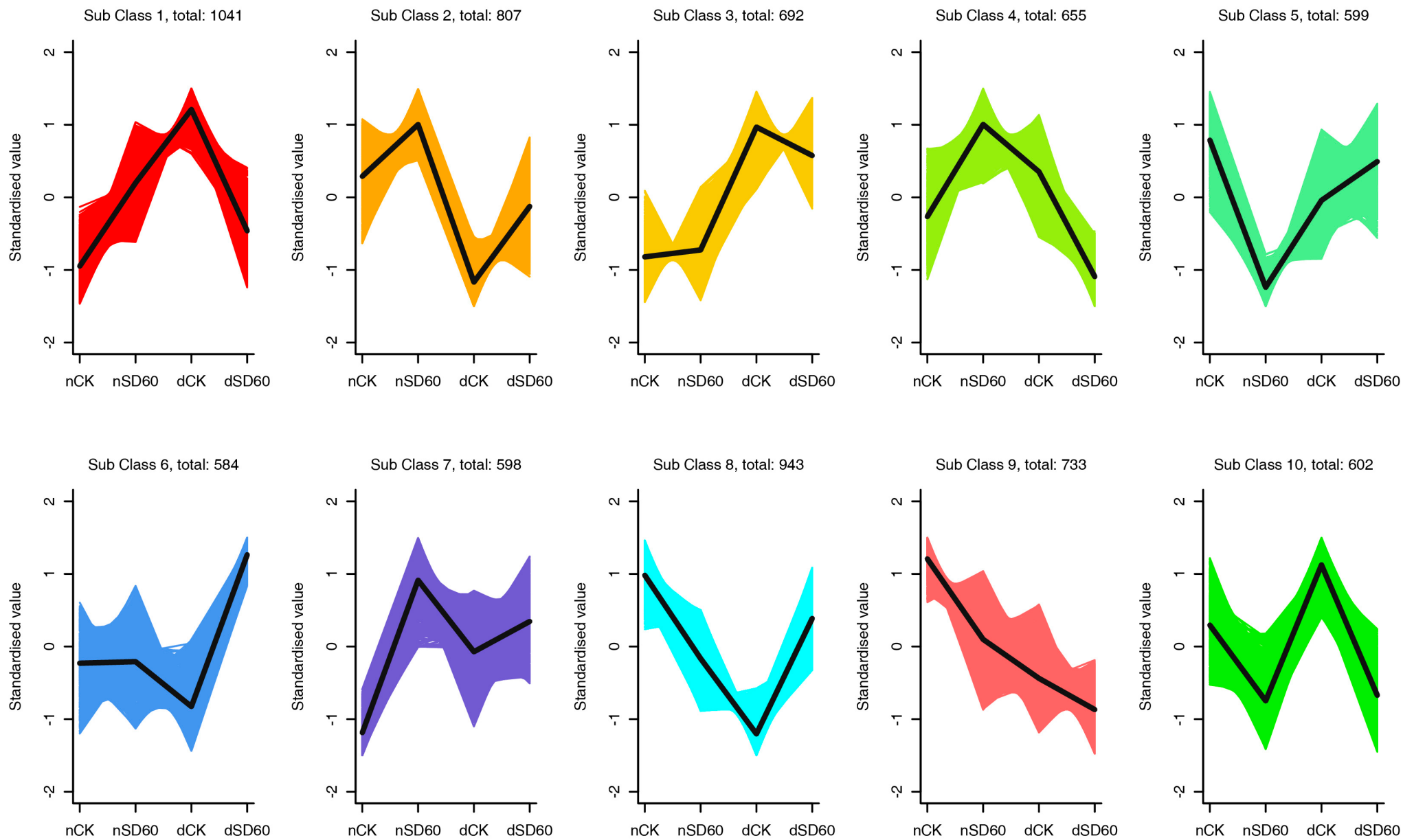

Supplement: Supplementary file 1 [file plants-13-00063-s001.zip › Figure S4.pdf]

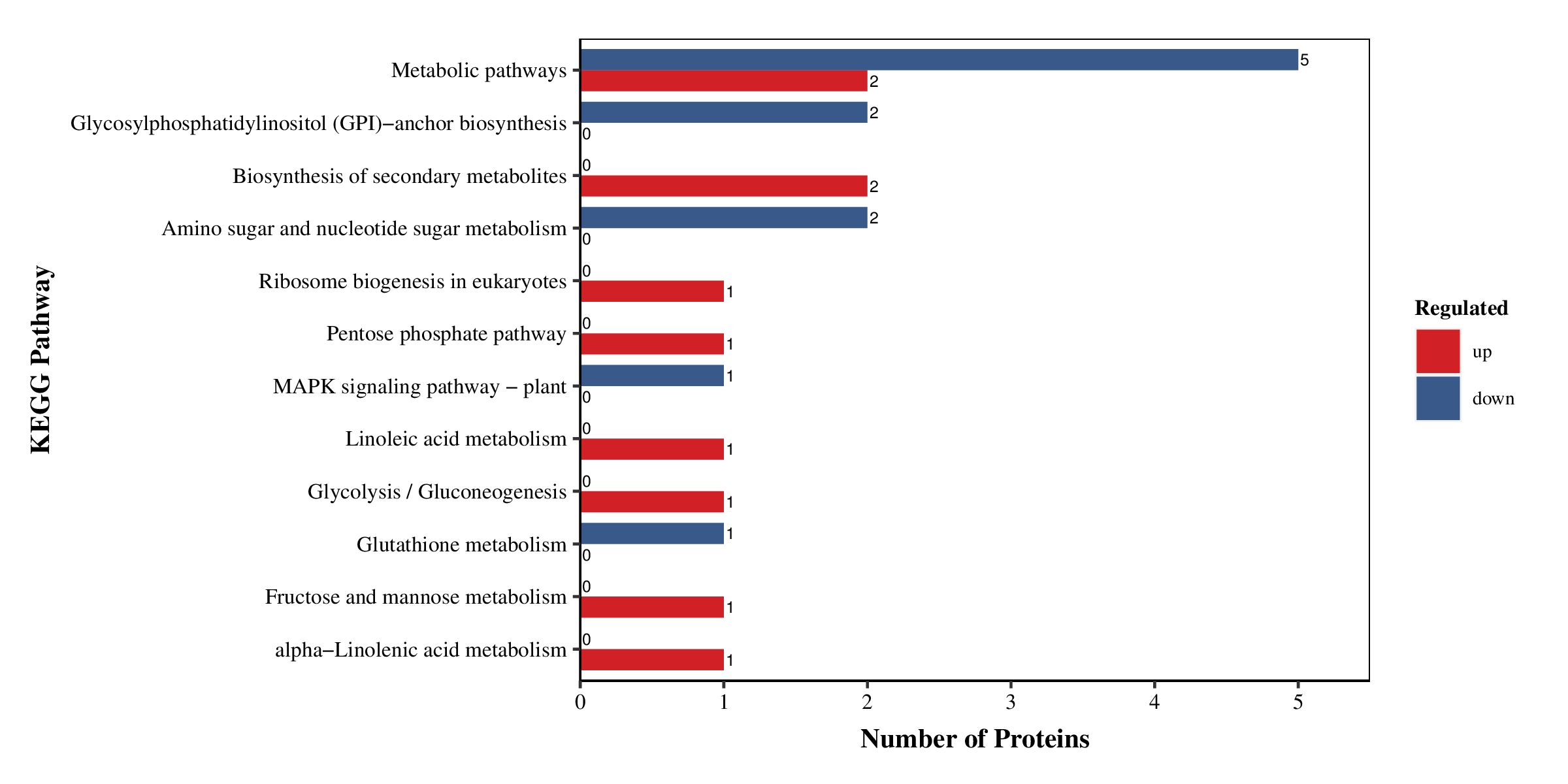

Supplement: Supplementary file 1 [file plants-13-00063-s001.zip › Table S3/dCK_vs_dSD60_KEGG_diff_bar.png]

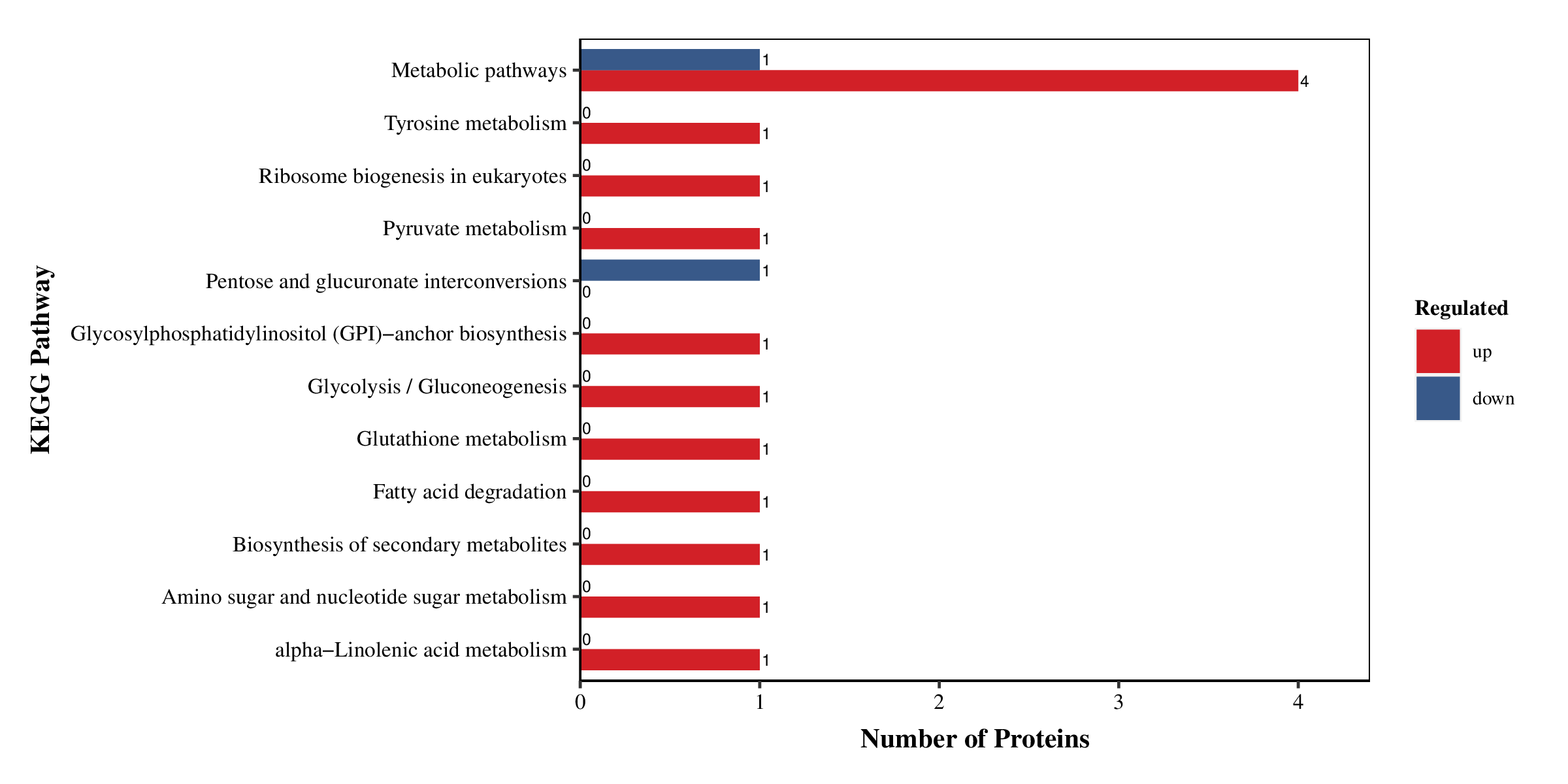

Supplement: Supplementary file 1 [file plants-13-00063-s001.zip › Table S3/nCK_vs_nSD60_KEGG_diff_bar.png]

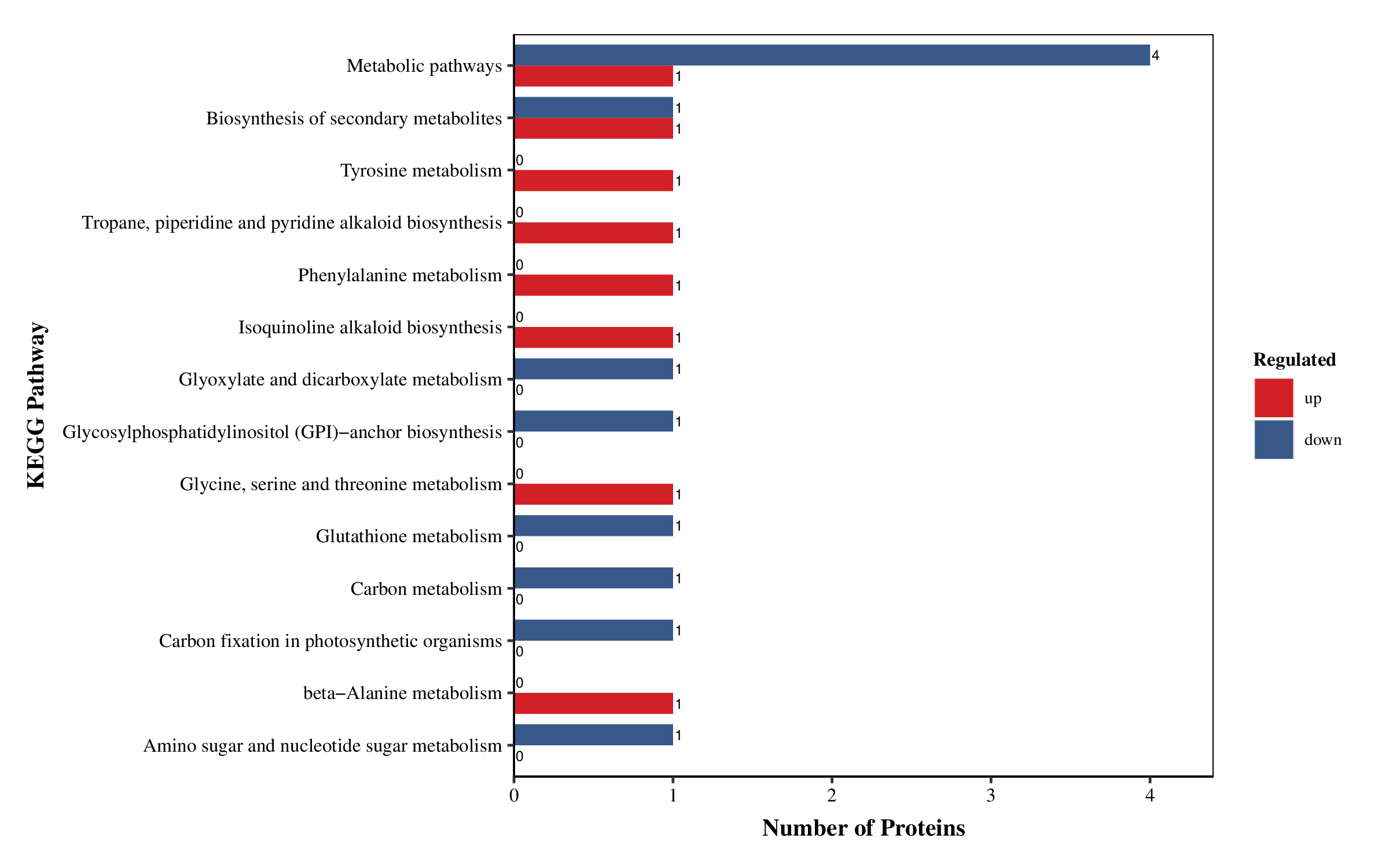

Supplement: Supplementary file 1 [file plants-13-00063-s001.zip › Table S3/nSD60_vs_dSD60_KEGG_diff_bar.png]
